# Supplementary material for: Effective control of SARS-CoV-2 transmission between healthcare workers during a period of diminished community prevalence of COVID-19
Source: eLife. 2020 Jun 19;9:e59391. doi: 10.7554/eLife.59391 (PMC7326489; doi:10.7554/eLife.59391)
Supplement: Transparent reporting form [file elife-59391-transrepform.docx]

***eLife’s* transparent reporting form**

We encourage authors to provide detailed information *within their submission* to facilitate the interpretation and replication of experiments. Authors can upload supporting documentation to indicate the use of appropriate reporting guidelines for health-related research (see [EQUATOR Network](http://www.equator-network.org/%20)), life science research (see the [BioSharing Information Resource](https://biosharing.org/)), or the [ARRIVE guidelines](http://www.plosbiology.org/article/info:doi/10.1371/journal.pbio.1000412) for reporting work involving animal research. Where applicable, authors should refer to any relevant reporting standards documents in this form.

If you have any questions, please consult our Journal Policies and/or contact us: [editorial@elifesciences.org](mailto:editorial@elifesciences.org).

**Sample-size estimation**

- You should state whether an appropriate sample size was computed when the study was being designed
- You should state the statistical method of sample size computation and any required assumptions
- If no explicit power analysis was used, you should describe how you decided what sample (replicate) size (number) to use

Please outline where this information can be found within the submission (e.g., sections or figure legends), or explain why this information doesn’t apply to your submission:

This was an observational study so sample size was not pre-determined.

**Replicates**

- You should report how often each experiment was performed
- You should include a definition of biological versus technical replication
- The data obtained should be provided and sufficient information should be provided to indicate the number of independent biological and/or technical replicates
- If you encountered any outliers, you should describe how these were handled
- Criteria for exclusion/inclusion of data should be clearly stated
- High-throughput sequence data should be uploaded before submission, with a private link for reviewers provided (these are available from both GEO and ArrayExpress)

Please outline where this information can be found within the submission (e.g., sections or figure legends), or explain why this information doesn’t apply to your submission:

This was an observational study; 360 individuals underwent repeat sampling as described in the results.

**Statistical reporting**

- Statistical analysis methods should be described and justified
- Raw data should be presented in figures whenever informative to do so (typically when N per group is less than 10)
- For each experiment, you should identify the statistical tests used, exact values of N, definitions of center, methods of multiple test correction, and dispersion and precision measures (e.g., mean, median, SD, SEM, confidence intervals; and, for the major substantive results, a measure of effect size (e.g., Pearson's r, Cohen's d)
- Report exact p-values wherever possible alongside the summary statistics and 95% confidence intervals. These should be reported for all key questions and not only when the p-value is less than 0.05.

Please outline where this information can be found within the submission (e.g., sections or figure legends), or explain why this information doesn’t apply to your submission:

Median was used to calculate the median TAT, raw data is in the table below for figure 1a.

Fisher’s exact test was used to compare the proportion of HCWs testing positive in this study period to that of our previous study period.

Pearson’s Chi-square test was used for comparison of the proportions of HCWs testing positive in each job role.

This can be found in the main text.

(For large datasets, or papers with a very large number of statistical tests, you may upload a single table file with tests, Ns, etc., with reference to sections in the manuscript.)

**Group allocation**

- Indicate how samples were allocated into experimental groups (in the case of clinical studies, please specify allocation to treatment method); if randomization was used, please also state if restricted randomization was applied
- Indicate if masking was used during group allocation, data collection and/or data analysis

Please outline where this information can be found within the submission (e.g., sections or figure legends), or explain why this information doesn’t apply to your submission:

Groups were assigned by route of entry into the screening programme: *HCW asymptomatic screening arm* or *HCW symptomatic screening arm* (including both symptomatic HCWs and symptomatic household contacts), as described in the methods.

**Additional data files (“source data”)**

- We encourage you to upload relevant additional data files, such as numerical data that are represented as a graph in a figure, or as a summary table
- Where provided, these should be in the most useful format, and they can be uploaded as “Source data” files linked to a main figure or table
- Include model definition files including the full list of parameters used
- Include code used for data analysis (e.g., R, MatLab)
- Avoid stating that data files are “available upon request”

Please indicate the figures or tables for which source data files have been provided:

Figure 1a and 1b Data: Source data file provided and data also shown below

| **Week commencing** | **Total tested** | **Total positive** | **Asymptomatics tested** | **Symptomatic tested** | **HHC tested** | **Unknown/ other tested** | **Asymptomatic positive** | **Symptomatic positive** | **HHC positive** | **Unknown/ other positive** |
| --- | --- | --- | --- | --- | --- | --- | --- | --- | --- | --- |
|  |  |  |  |  |  |  |  |  |  |  |
| 06-Apr-20 | **143** | **6** | 121 | 15 | 7 | 0 | 4 | 1 | 1 | 0 |
| 13-Apr-20 | **463** | **37** | 383 | 60 | 18 | 2 | 20 | 14 | 3 | 0 |
| 20-Apr-20 | **663** | **18** | 529 | 95 | 26 | 13 | 7 | 11 | 0 | 0 |
| 27-Apr-20 | **731** | **18** | 550 | 119 | 62 | 0 | 11 | 7 | 0 | 0 |
| 04-May-20 | **641** | **12** | 483 | 104 | 50 | 4 | 8 | 3 | 1 | 0 |
| 11-May-20 | **954** | **1** | 738 | 164 | 51 | 1 | 1 | 0 | 0 | 0 |
| 18-May-20 | **1062** | **3** | 840 | 168 | 53 | 1 | 1 | 2 | 0 | 0 |

Figure 1c and 1d Data: Source data file provided and data also shown below

| Week commencing | Positive patient tests in CUHNFT | Positive patient tests in EOE |
| --- | --- | --- |
| 06-Apr-20 | 94 | 2292 |
| 13-Apr-20 | 48 | 1840 |
| 20-Apr-20 | 36 | 1842 |
| 27-Apr-20 | 38 | 1482 |
| 04-May-20 | 32 | 1000 |
| 11-May-20 | 18 | 681 |
| 18-May-20 | 6 | 417 |
